# Transcriptome and Metabolome Analyses Reveal the Regulatory Mechanism of *TC1a* in the Sucrose and Starch Synthesis Pathways in *Arabidopsis thaliana*

**DOI:** 10.3390/plants13233402

**Published:** 2024-12-04

**Authors:** Wenjun Zhu, Guangze Li, Han Shi, Ying Ruan, Chunlin Liu

**Affiliations:** 1 Yuelushan Laboratory, Hunan Agricultural University, Changsha 410128, China; 17809972112@163.com (W.Z.); 15379295903a@gmail.com (G.L.);; 2 Key Laboratory of Hunan Provincial on Crop Epigenetic Regulation and Development, Hunan Agricultural University, Changsha 410128, China

**Keywords:** *Arabidopsis thaliana*, phylogenetic analysis, TRAF proteins, sucrose and starch metabolic pathways, transcriptomics and targeted metabolomics analysis

## Abstract

Tumor necrosis factor receptor-associated factor (TRAF) proteins, originally identified in mammals, have since been found in most plants. TRAF proteins in plants have been shown to be involved in cellular autophagy, immunity, drought resistance, and ABA induction. However, the role in regulating sucrose and starch metabolism has not been reported. In this study, we confirmed that *TC1a* can regulate sucrose and starch metabolism through gene editing, phenotypic observation, transcriptomics and metabolomics analyses. Initially, 200 and 81 TRAF proteins were identified in rapeseed (*Brassica napus* L.) and *Arabidopsis thaliana*, respectively, and divided into five classes. We found that overexpression of *TC1a* inhibited root length, plant height, flowering, and leaf development in *A. thaliana*. Additionally, 12 differentially expressed genes (DEGs) related to sucrose and starch metabolism pathways were identified in overexpressing and knockout plants, respectively. Six differentially accumulated metabolites (DAMs)—fructose, sucrose, glucose, trehalose, maltose, and 6-phosphate fructose—were identified using widely targeted metabolomics analysis. The results show that *TC1a* affects the growth and development of *Arabidopsis*, and induces the expression of sucrose and starch synthase and hydrolases, providing a foundation for further research into its molecular mechanisms.

## 1. Introduction

Sucrose, a natural, non-reducing disaccharide, is primarily found in photosynthetic organisms such as unicellular algae, blue-green algae and higher plants [1,2]. As the primary photosynthesis product, sucrose is vital for plant signaling, environmental stress adaptation, and processes like germination, development, flowering, and senescence, and influences biological processes such as seed germination, seedling development, flowering and senescence [1,3,4,5]. It is also contributes to the synthesis of anthocyanins, starch, fructans and storage proteins [6,7,8]. Sucrose synthesis mainly occurs in source and sink tissues, where Calvin cycle-generated triose phosphate is transported from chloroplasts to the cytoplasm [8]. Its synthetic pathway is catalyzed by sucrose-phosphate synthase and sucrose 6′-phosphate phosphatase. Sucrose metabolism, a key plant metabolic pathway, is essential for carbon allocation and sugar signaling [3,9]. It is decomposed into glucose and fructose by sucrose hydrolase. Fructose and uridine diphosphate glucose (UDP-glucose) can be synthesized through the reverse catalytic activity of sucrose synthase [3,9,10]. Sucrose, synthesized in leaves during photosynthesis, is stored at high concentrations in sieve tubes and fluctuates between day and night [11,12,13]. Sucrose depends on phloem loading and unloading for its long-distance transportation in plants, synthesis in mesophyll cells and storage in vacuoles. It is transported to the bundle sheath (BSC) and phloem parenchyma (PP) via the protoplastid and symplastid pathways [14,15]. Subsequently, it is conveyed to the sieve element/companion cell complex (SE/CC) and ultimately transported via the sieve tube (ST) to the sink tissues through osmotic pressure, and released from the phloem to supply carbon sources for plants.

Starch mainly exists in flowers, seeds, leaves and other organs, and is synthesized in plastids and stored in chloroplasts through photosynthesis; however, for long-term storage, it is located in the amyloplast [16]. In Arabidopsis, 30–50% of the assimilates produced by the Calvin cycle are allocated to starch synthesis [17]. During starch synthesis, fructose 6-phosphate is converted into glucose 1-phosphate (Glu1P) by phosphoglucoisomerase (PGI) and phospho-glucomutase (PGM1). Glu1P and ATP synthesize adenosine diphosphate glucose (ADPGlc) under the action of ADP-glucose pyrophosphorylase (AGPase). Ultimately, amylopectin, or amylose, is synthesized by granular-bound starch synthase (GBSS), soluble starch synthase (SSs), and starch branching enzyme (SBE) and starch debranching enzyme (DBE) [18]. The main products of starch breakdown are maltose and glucose. At night, starch glucose residues are phosphorylated by glucan water dikinase (GWD) and phosphoglucan water dikinase (PWD) to break down into glucan. These glucans are subsequently degraded into glucose and maltose by α-amylase, β-amylase and hydrolytic enzymes [18]. This process supplies carbon skeletons for synthesizing other compounds and facilitates both sucrose synthesis and degradation [19].

Sucrose and starch are the primary products of plant photosynthesis [20]. Their distribution is influenced by carbohydrate storage in leaves and the diurnal pattern of their transfer to sink tissues [21]. They play regulatory roles in plant growth and development, and responses to hormones and abiotic stress. The sucrose–starch metabolic pathways are complex. Through coordinating the synthesis and decomposition of the two carbohydrates, carbon allocation in photosynthesis can be effectively regulated, which can improve the effective utilization of photosynthetic products in plants [22]. A new molecular model regulating the coordination of starch and sucrose in barley was recently proposed. *SUSIBA1* and *SUSIBA2* are fructooligosaccharide inhibitory factors and starch synthesis-activating factors encoded by a single gene dual promoter. When sucrose content is low, *SUSIBA1* expression is enhanced while *SUSIBA2* expression is inhibited, leading to reduced starch and fructooligosaccharide production. In contrast, when sucrose content increases, the contents of starch and fructan also increase [22]. Starch synthesis is primarily catalyzed by AGPase. Overexpression of *APL1* in *Vitis vinifera* starch hydrolases *AMY1*, *BAM1*, *BAM3* and *BAM8* is significantly up-regulated. Additionally, starch conversion to soluble sugars can regulate osmotic balance under low-temperature conditions [23].

TRAF proteins function as molecular adapters or E3 ubiquitin ligases to regulate downstream substrate signal transduction [24,25,26]. Meprins, specific metalloproteases of the astacin family [27], possess a highly conserved TRAF domain, also known as the meprin and TRAF-C homology (MATH) domain. The unique feature of TRAF proteins is the C-terminal domain, which is divided into TRAF-N and TRAF-C domains. The TRAF-C domain consists of 180 amino acids and forms 7 to 8 antiparallel β-sheets [26,28]. Proteins with MATH domains primarily participate in individual development and cell differentiation in humans [29,30] and animals [24,31,32]. Numerous MATH domain proteins have also been identified in plants, including *A. thaliana* [33], *Brassica rapa* [34], maize [35], *Oryza sativa* [36] and *Solanaceae* spp. [37]. Phylogenetic analysis indicates that the TRAF protein family in animals is divided into eight subfamilies [38]. In *A. thaliana* and rice, TRAF proteins are grouped into four subgroups: MATH-only proteins, MATH-BPM proteins, MATH-UBP proteins, and MATH-PEARLI-4 proteins [39]. Additionally, in rapeseed, TRAF proteins are categorized into six classes, labeled as Class I to Class VI [34]. The absence of *MUSE13/MUSE14* in *Arabidopsis* can lead to the accumulation of NLR and autoimmunity because *MUSE13*/*MUSE14* formed TRAFasome complexes with SCFCPR1 and NLRs, regulated NLR ubiquitination and negatively regulated plant immune response [40]. Another study reported that *TRAF1* interacts with TRAF2 to regulate TNFR2 signaling transduction and neuronal cell death. Specifically, TRAF1a (*MUSE13*) and TRAF1b (*MUSE14*) interacted with ATG6, SINAT1, SINAT2 and SINAT6 to regulate the autophagy process [41]. MATH-BTB domain proteins are highly conserved throughout evolution but exhibit significant genome expansion in rice [42]. BTB/POZ-MATH (BPM) proteins directly interact with ABA to negatively regulate *AtHB6*, which is an HD-ZIP transcription factor activator that negatively regulates the expression of *Rab18*, *RD22* and *RD29B*. SINA proteins in plants regulate lateral root development [43,44] and drought stress resistance [45]. Although SINA2 lacks the RING domain, it is highly homologous to SINAs and contains an ARBE motif in the promoter sequence. Therefore, ABRE-binding proteins AREB1 and AREB2 regulate the expression of SINA2, and positively regulate drought resistance. BPM proteins are essential for plant development, and their functional loss can affect both fatty acid synthesis and metabolism. Based on the E3 ligase CULLIN3 mediated by MATH-BTB proteins, it can interact with a large number of ERF/AP2 transcription factors. Furthermore, BPMs negatively regulate *WRI1* for fatty acid synthesis, thereby affecting plant fatty acid content. *AtUPB12* and *AtUPB13* interact with the ATG6 and E3 ligases SINAT1, SINAT2, and SINAT6 to influence leaf and root development and participate in the regulation of flowering and circadian rhythms [46,47]. SINA3 mediates the degradation of NAC transcription factors through ubiquitination. The TRAFCANDIDATE 1b (*TC1b*) mutant, screened in the background of the suppressor of npr1-1, conservative 1 (snc1), inhibits the phenotype [48].

Although numerous functions of TRAF proteins have been characterized in plants, how the functions of sucrose and starch metabolism can be influenced has not yet been investigated. Therefore, we created CRISPR/Cas9 and overexpression Arabidopsis mutants of *TC1a*; integrated the analysis of transcriptome, metabolome, and phenotypic characteristics; and systematically analyzed their potential regulatory mechanisms in regulating sucrose and starch metabolic pathways in Arabidopsis.

## 2. Results

### 2.1. Classification and Identification of TRAF Proteins in Arabidopsis and Rapeseed

**Figure 1:**
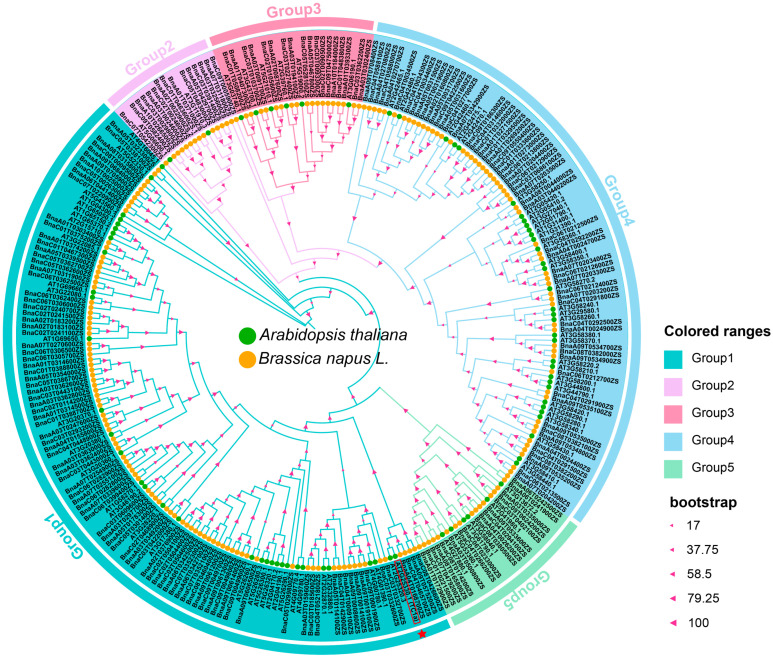
Phylogenetic analysis of TRAF proteins in *Arabidopsis* and rapeseed. The yellow and green circles represent the TRAF proteins of *Arabidopsis* and rapeseed, respectively. *AtTC1a* (*At2G25330.1*) has been labeled with rectangle and red star.

In this study, we identified 281 TRAF proteins, including 81 in *Arabidopsis* and 200 in rapeseed. These proteins were classified into five groups: MATH-only proteins, MATH-BPM proteins, MATH-UBP proteins, MATH-PEARLI-4 proteins, and SINA proteins. In *Arabidopsis* and rapeseed, the numbers of family members for MATH-only proteins, MATH-BPM proteins, MATH-UBP proteins, MATH-PEARLI-4 proteins, and SINA proteins are 58 and 145, 6 and 18, 2 and 11, 9 and 8, and 6 and 18, respectively. The MATH-only protein subfamily was further divided into four categories based on the number of MATH domains: one, two, three, or four domains. Among these categories, there are 30 and 88 members with one MATH domain, 26 and 51 with two domains, and 2 and 4 with four domains in Arabidopsis and rapeseed, respectively. Interestingly, only two members in rapeseed were found to contain three MATH domains. *AtTC1a* (*At2G25330.1*), which contains four tandem MATH domains, belongs to Group 1, and is found exclusively in *Arabidopsis*, with no homologous genes identified in rapeseed (Figure 1).

### 2.2. GUS Staining and RT-qPCR Analysis

**Figure 2:**
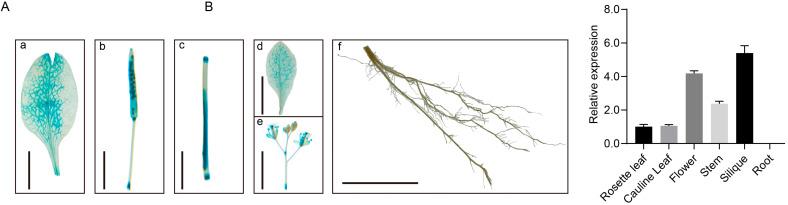
Histochemical staining and expression profile analysis. (**A**) GUS staining (a: rosette leaf, b: silique, c: stem, d: cauline leaves, e: flower, f: root). a–e: scales are 0.5 cm, f: scale is 1 cm. (**B**) RT-qPCR analysis.

To explore the expression of *TC1a* in different tissues of Arabidopsis, a histochemical staining method was employed. The results showed that *TC1a* was highly expressed in siliques and inflorescences, moderately expressed in leaves and stems, and barely expressed in roots (Figure 2A). These results are consistent with the expression data from the TAIR database. The RT-qPCR analysis of expression levels in different tissues was consistent with the GUS staining results (Supplementary Table S4.1, Figure 2B).

### 2.3. Obtaining Homozygous Transgenic Arabidopsis thaliana

**Figure 3:**
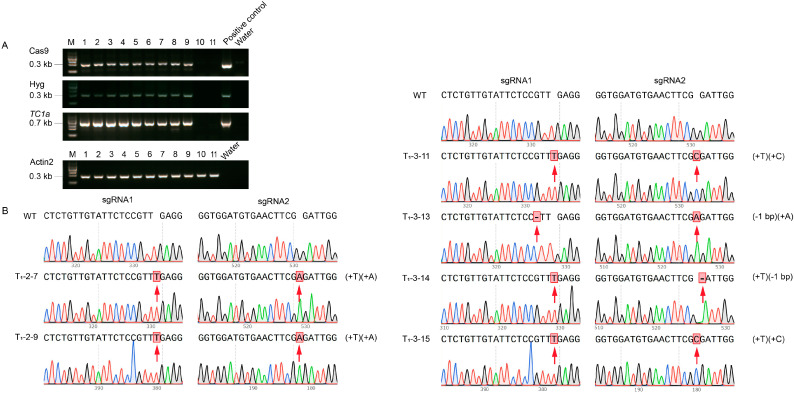
T_1_ generation sequencing analysis of transgenic *Arabidopsis thaliana*. (**A**) Detection of Cas9 protein, HYG resistance gene and *TC1a* expression frame. (**B**) Monoclonal sequencing analysis of *TC1a #2* and *TC1a #3*. Red arrows indicate insertion or deletion sites.

**Figure 4:**
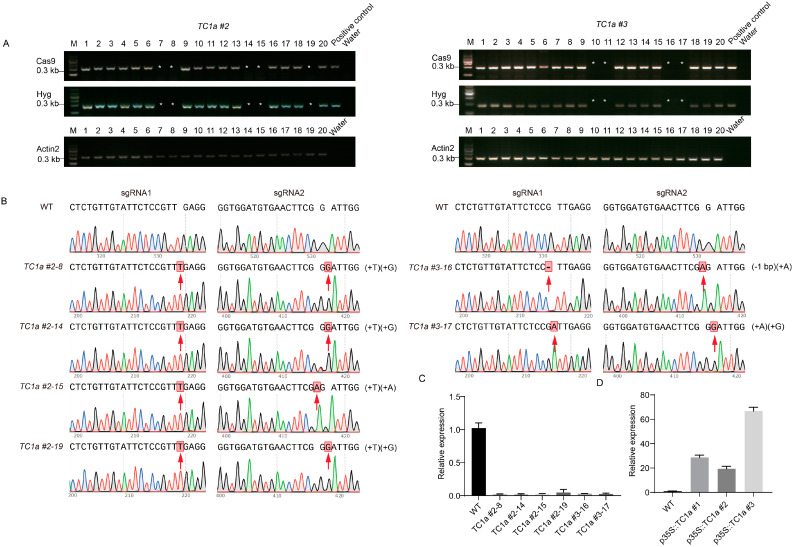
T_2_ generation gene editing and transcription level detection of overexpressing plants. (**A**) Identification of Cas9 protein and HYG resistance genes in T_2_ of *TC1a #2* and *TC1a #3*, with the symbol * indicating that Cas9 and HYG have been removed from the transgenic materials or non-transgenic materials. (**B**,**C**) Detection of editing types and gene transcription levels in homozygous *Arabidopsis thaliana*. (**D**) Analysis of gene expression levels in overexpressing plants.

Transgenic plants were identified by amplifying the Cas9 protein, the HYG resistance gene, and the expression cassette of *TC1a* (Figure 3A). The target sequence was amplified through PCR and subsequently sequenced to identify *TC1a #2* and *TC1a #3*, which displayed distinct editing types. In *TC1a #2*, T and A bases were inserted into sgRNA1 and sgRNA2, respectively. *TC1a #3* produced three types of editing: the first inserted T and C into sgRNA1 and sgRNA2, respectively; the second inserted A into sgRNA2 with a single base deletion in sgRNA1; and the third inserted T into sgRNA1 with a single base deletion in sgRNA2 (Figure 3B). In order to obtain homozygous strains, *TC1a #2* and *TC1a #3*, which had been successfully edited in the T_1_ generation, were harvested. From each plant, 20 strains were selected for Cas9 protein and HYG amplification analysis. *TC1a #2-7*, *TC1a #2-8*, *TC1a #2-14*, *TC1a #2-15*, *TC1a #2-19*, *TC1a #3-10*, *TC1a #3-11*, *TC1a #3-16* and *TC1a #3-17* successfully removed plasmid vectors (Figure 4A). Then, it was found using PCR purification solution sequencing that only *TC1a #2-7*, *TC1a #3-10* and *TC1a #3-11* were not edited. As a result, six homozygous plants were obtained (Figure 4B), and the expression level of *AtTC1a* was detected (Supplementary Table S4.2). The mutants with almost no expression were selected as the subsequent experimental materials (Figure 4C).

*p35S::TC1a* transgenic plants were generated through herbicide resistance screening. The transcription level was analyzed using RT-qPCR (Supplementary Table S4.3), and resistance screening continued until T_2_ and T_3_ homozygotes were selected for subsequent experimental materials (Figure 4D).

### 2.4. Phenotypic Observation and Analysis

**Figure 5:**
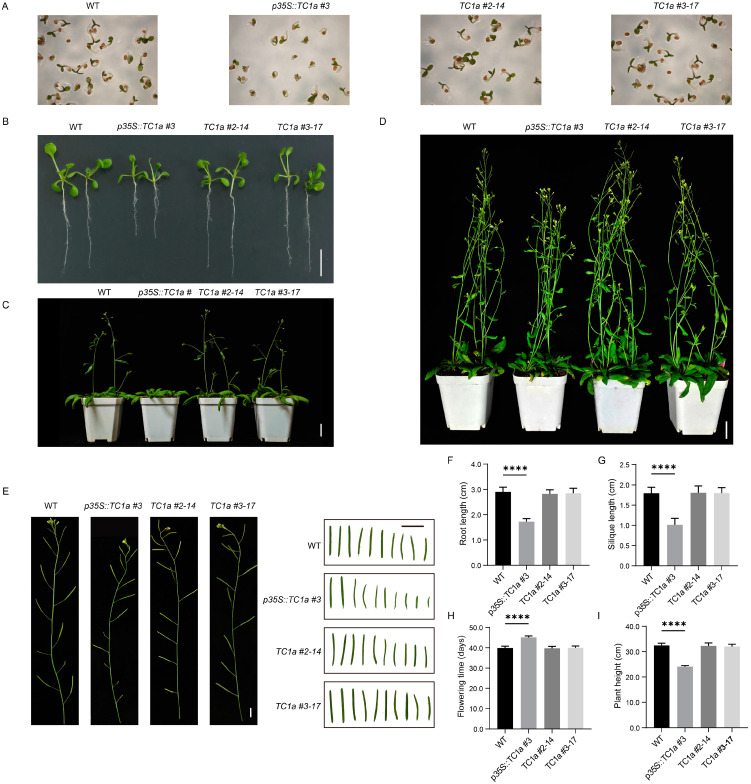
Phenotypic observation and statistics. (**A**) Seed germination test. (**B**–**E**) Photographic observation of root length, flowering, plant height, siliques and stems. (**F**–**I**) Data from statistical analysis (n = 20). The symbol **** indicates a statistically significant deviation from WT at *p* < 0.0001 probability levels. (**B**,**E**) Bars for 1 cm. (**C**,**D**) Bars for 2 cm.

Compared to WT, the overexpressing lines showed significantly slower germination rates and reduced germination speed (Figure 5A). After 10 days of germination, the root length was reduced to approximately two-thirds of that in WT. At the 10-leave stage, the length, width and area of the 3th, 5th and 7th leaves were significantly reduced, while there were no significant differences in the knockout lines (Figure 5B,F and Figure S3). During the reproductive growth stage, the knockout plants bolted and flowered at day 40, similar to WT, while the overexpressing lines exhibited a delayed flowering period, occurring between days 44–46 (Figure 5C,H). At the beginning of the ripening stage, the siliques and plant height of overexpressing plants were significantly shorter than those of WT (Figure 5D,E,G,I). Detailed phenotypic data are provided in Supplementary Table S5.

In summary, overexpression of *TC1a* significantly inhibited the growth and germination of roots, siliques, leaves and plant height, while also delaying flowering. The dwarf phenotype was strikingly similar to the *TC1b* mutants. Consequently, overexpression of *TC1a* negatively regulated the growth and development of *Arabidopsis*.

### 2.5. Determination and Analysis of Sugar Content

**Figure 6:**
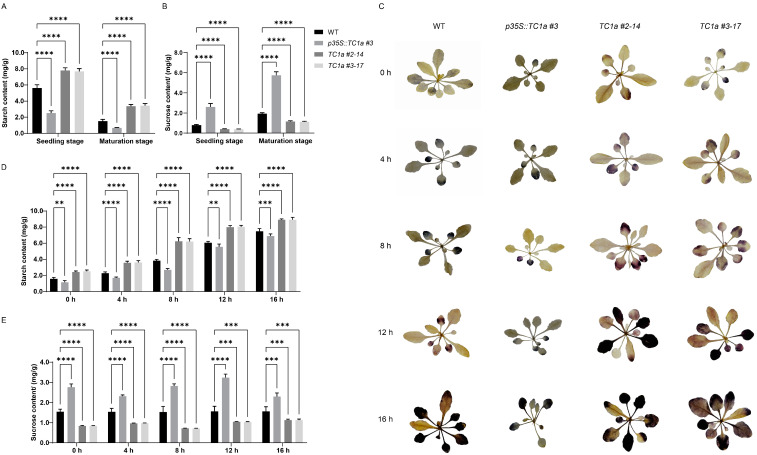
Sucrose and starch content determination and I_2_-IK staining of transgenic Arabidopsis and WT. (**A**,**B**) Analysis of starch and sucrose contents at seedling and maturation stages (n = 10). (**C**) Starch staining; the scale is 2 cm. (**D**,**E**) Starch and sucrose content determination (n = 10). The symbols **, *** and **** indicate a statistically significant deviation from WT at *p* < 0.01, *p* < 0.001 and *p* < 0.0001 probability levels.

The starch and sucrose contents in the leaves were measured at the seedling and maturation stages (Supplementary Table S6). The results showed that the starch content was significantly reduced, while the sucrose content was significantly increased in the overexpressing lines, in contrast to the knockout lines (Figure 6A,B). Therefore, we hypothesized that *TC1a* may play a crucial role in the sucrose and starch metabolism in *Arabidopsis*.

To further investigate whether *TC1a* is involved in sucrose and starch metabolism, we analyzed the changes in sucrose and starch contents after exposing the plants to different durations of light. After exposing the plants to light for 0 h, 4 h, 8 h, 12 h and 16 h, starch staining was performed. Both WT and mutant materials showed a gradual increase in starch accumulation with prolonged light exposure. Compared to WT, the overexpressing lines exhibited lighter staining, while the knockout lines showed darker staining overall (Figure 6C).

After light exposure, the starch and sucrose contents in the leaves were measured (Supplementary Table S7). The starch content trend aligned with I_2_-KI staining results. Overexpressing lines accumulated less starch, while knockout lines accumulated more starch than WT (Figure 6D). The sucrose content was significantly higher in overexpressing lines and significantly lower in knockout lines compared to WT (Figure 6E). With extended light exposure, the sucrose content in overexpressing lines initially decreased, then increased, followed by another decrease, peaking at 12 h. In knockout lines, the sucrose content initially increased, then decreased, and increased again, reaching a maximum at 16 h. These findings indicate that *TC1a* plays a role in the starch and sucrose metabolism process.

### 2.6. Transcriptomic and Metabolomic Analysis

**Figure 7:**
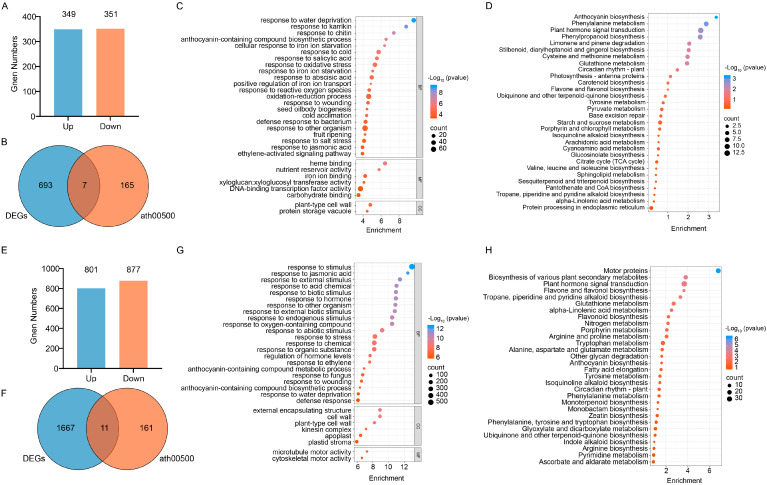
Transcriptome and metabolome analysis. (**A**–**D**) and (**E**–**H**) DEGs up-regulated and down-regulated numbers, Venn plot analysis between DEGs and ath00500 pathway-related genes, GO enrichment analysis, KEGG annotation in WT vs TC1a and WT vs TC1a-OE, respectively.

To further confirm the role of *TC1a* in sucrose and starch metabolism, transcriptome sequencing identified 700 DEGs in *TC1a-OE*, including 349 up-regulated and 351 down-regulated (Figure 7A). In *TC1a* knockout lines, 1678 DEGs were identified, with 801 up-regulated and 877 down-regulated genes (Figure 7E). Visualization of the top 30 GO terms revealed that the biological processes (BPs) in knockout lines primarily included response to jasmonic acid, ethylene, and endogenous stimuli, as well as regulation of hormone levels, stress responses, and anthocyanin biosynthesis. Cellular components (CCs) included the cell wall, kinesin complex, apoplast, and plastid stroma. Molecular functions (MFs) encompassed microtubule motor activity and cytoskeletal motor activity (Figure 7G). In *TC1a-OE* lines, BPs primarily involved responses to salicylic acid, abscisic acid, water deprivation, and salt stress, along with anthocyanin biosynthesis. CCs included plant-type cell wall, and protein storage vacuole. MFs included xyloglucan, xyloglucosyl transferase activity and DNA-binding transcription factor activity, etc. (Figure 7C). KEGG pathway analysis showed that pathways related to glucose metabolism in *TC1a-OE* included starch and sucrose metabolism, glucosinolate biosynthesis, pyruvate metabolism, and citrate cycle (TCA cycle) (Figure 7D). The pathways related to glucose metabolism in knockout materials included plant hormone signal transduction, anthocyanin biosynthesis, and other biosynthetic pathways (Figure 7H).

Seven DEGs (*BAM6*, *GH9C3*, *SPS4F*, *BGLU4*, *BGLU19*, *BAM5*, *TPPH*) and 11 DEGs (*BGLU33*, *TPPH*, *TPS9*, *BGLU1*, *ISA3*, *BAM5*, *FRK1*, *BGLU11*, *APL3*, *PYK10*, *BGLU22*) related to starch and sucrose metabolic pathways were identified in *TC1a* and *TC1a-OE*, respectively. In *TC1a-OE*, *BAM5*, *BAM6*, *BGLU19*, *BGLU4*, *CH9C3* and *SPS4F* were significantly up-regulated, whereas TPPH was significantly down-regulated (Figure S4A). In WT_vs_TC1a, starch hydrolases *BAM5*, *ISA3*, *BGLU1*, *BGLU11*, *BGLU22*, *PYK10*, *FRK1* and *APL3* were significantly up-regulated, whereas *BGLU33*, *TPPH* and *TPS9* were significantly down-regulated (Figure 5A).

A total of 19 metabolites were detected in WT_vs_TC1a-OE and 5 in WT_vs_TC1a (Table 1). The DAMs related to starch and sucrose metabolism included sucrose, trehalose, fructose, 6-phosphate fructose, glucose and maltose. OPLS-DA analysis clearly distinguished the metabolite differences between *WT*, *TC1a*, and *TC1a-OE* (Figure S3).

Cluster analysis revealed that the sucrose content in *TC1a* was significantly lower than in WT, whereas trehalose, fructose, glucose and maltose were significantly higher (Figure S5B). In *TC1a-OE*, trehalose, fructose and glucose were significantly lower, whereas sucrose, maltose and 6-phosphate fructose were significantly higher (Figure S4B). Correlation analysis between sucrose and starch related DEGs and DAMs showed a significant positive correlation between *SPS4F* expression and sucrose content in *TC1a-OE* (Figure S4C). In *TC1a*, the starch synthase *APL3* was significantly positively correlated with the content of glucose and maltose. The results suggest that the changes in sucrose and starch contents in mutants may result from abnormal expression of related genes (Figure S5C).

### 2.7. Transcriptome and Metabolome Data Were Validated and Analyzed with RT-qPCR

**Figure 8:**
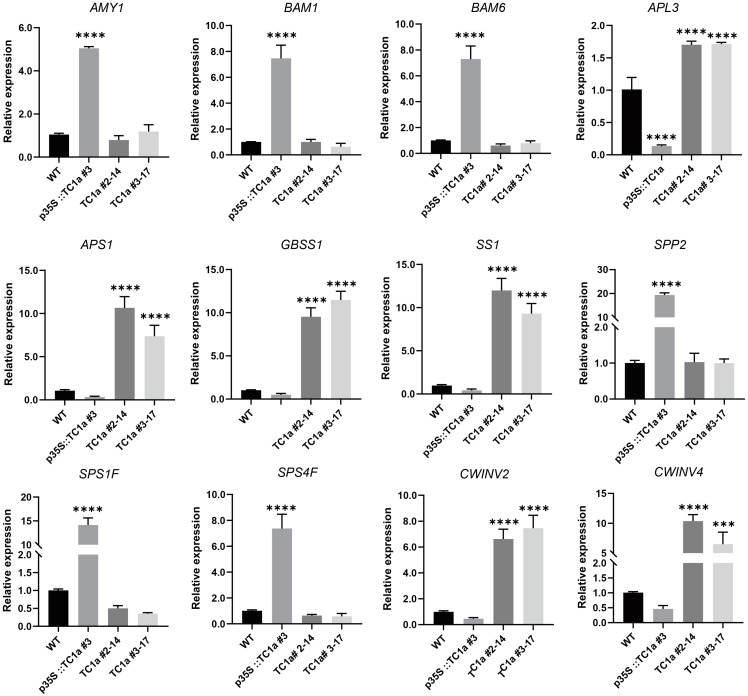
Analysis of the expression levels of key sucrose and starch genes in WT, overexpression and knockout lines using RT-qPCR. The symbol *** and **** indicates a statistically significant deviation from WT at *p* < 0.001 and *p* < 0.0001 probability levels.

**Figure 9:**
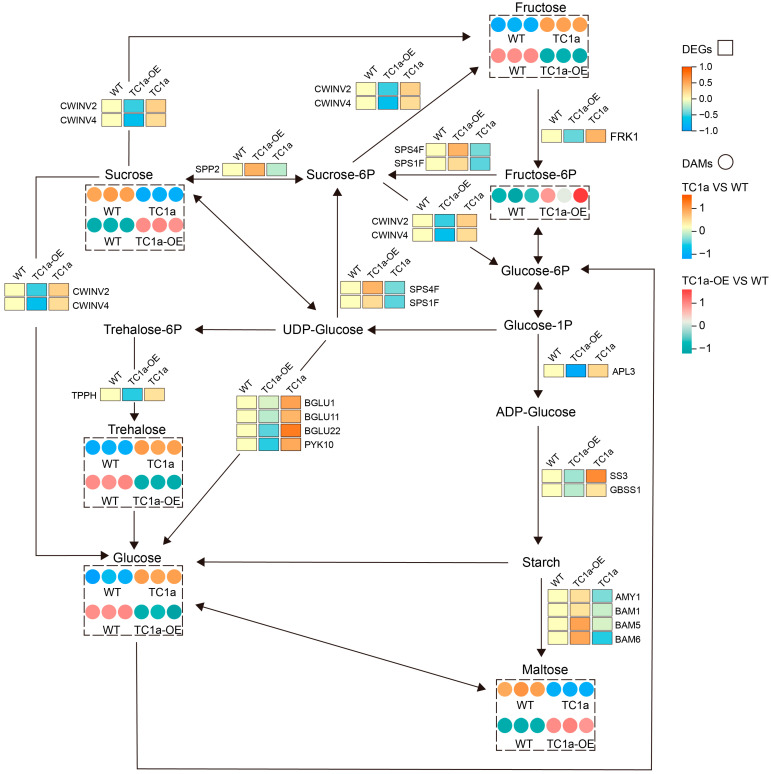
Analysis of sucrose starch synthesis metabolic pathway; data were normalized by z-score. Rectangles and circles represent DEGs and DAMs, respectively.

To investigate the regulation of *TC1a* in the starch and sucrose metabolic pathway, 12 genes related to starch and sucrose synthesis and hydrolysis were selected for RT-qPCR analysis (Supplementary Table S4.4). The results indicated that the expression levels of sucrose synthase and starch hydrolase were significantly increased, whereas those of sucrose hydrolase and starch synthase were significantly decreased in the overexpressing lines (Figure 8 and Figure 9); additionally, *APS1*, *APL3*, *SS1* and *GBSS1* showed significant up-regulation in the knockout plants. *BAM1*, *BAM5*, and *AMY1* were significantly up-regulated in overexpressing plants, resulting in a decrease in starch content, while starch accumulation was higher in the knockout lines. *SPS1F*, *SPS4F*, and *SPP2* were significantly up-regulated in overexpressing plants. *CWINV2* and *CWINV4* were significantly up-regulated in the knockout mutants.

## 3. Discussion

Previous studies have demonstrated that TRAF proteins are categorized into eight subfamilies: USP7, MATHd/RluA, MATHd-only, MATHd/BTB, MATHd/filament, TRAF, TRIM37, and Meprin families [38]. A recent study classified Arabidopsis TRAF proteins into five categories: MATH-only, MATH-BPM, MATH-UBP, MATH-PEARLI-4, and SINA subfamilys [39]. *Arabidopsis thaliana* encodes six SINA proteins (*AtSINA1*–*AtSINA6*). Among them, *AtSINA1*–*AtSINA4* contain RING and zinc finger domains, whereas *AtSINA5* and *AtSINA6* lack these domains. Hua Qi et al. identified 71 proteins in *Arabidopsis* [39]. In comparison, our study identifies 10 additional members of the TRAF protein family. Seven genes contain a single MATH domain (*AT1G31390*, *AT1G31400*, *AT2G01790*, *AT2G05410*, *AT2G05420*, *AT2G42475*, and *AT3G58370*). Two genes (*AT2G42460* and *AT2G42465*) contain the MATH-PEARLI-4 domain, while *AT3G22085* encodes two MATH domains. Phenotypic observations revealed that TC1a overexpression caused delayed germination, reduced root length, delayed flowering, smaller leaves and siliques, and shorter plant height. Additionally, *TC1a* overexpression exhibited a dwarfing phenotype similar to overexpression of *TC1b* in *snc1* [48] (Figure 5). Furthermore, *TC1a* overexpression resulted in excessive sucrose accumulation and abnormal starch consumption, whereas knockout lines showed increased sucrose consumption and starch accumulation (Figure 6). Combined transcriptome and metabolome analyses showed that both *TC1a* overexpression and knockout altered the expression of genes in sucrose and starch metabolism pathways in *Arabidopsis*, resulting in abnormal metabolite accumulation and consumption (Figure 7 and Figure 8). Therefore, *TC1a* potentially regulates the sucrose starch metabolic pathway.

Several TRAF proteins that regulate plant growth and development have been identified. Overexpression of *BPM1* cause curling and shrinking of *Arabidopsis* leaves [49]. The *ubp12-2w* mutants generated by T-DNA insertion exhibit dwarfism, early flowering, and multiple branching phenotypes [50]. *UBP12*/*UBP13* acted upstream of *DA1*, *DAR1* and *DAR2*, which negatively regulate plant leaf size [47]. *TC1a* and *TC1b* are the only TRAF proteins in *Arabidopsis* with four MATH domains. T-DNA insertion mutations in the first and fourth exons of *TC1a* and *TC1b*, respectively, produced transgenic lines (*tc1a-1*/*tc1b-1*). These lines were hybridized with *scn1* to create *tc1a-1 snc1* and *tc1b-1 scn1*. The morphology of *tc1a-1 scn1* resembled *scn1*; however, *tc1b-1 scn1* exhibited the opposite phenotype characterized by a significantly larger plant. The *TC1a*/*TC1b* co-knockout lines were generated, displaying phenotypes consistent with *tc1b-1 scn1*. Overexpression of *TC1b* exacerbated the dwarf phenotype of *scn1* [48]. Here, we successfully generated knockout and overexpression homozygous mutants. Overexpression lines exhibited inhibited growth and development, delayed germination, postponed bolting and flowering, and significantly smaller leaf size (Figure 5 and Figure S2). The morphological characteristics of the knockout lines were consistent with WT.

The experiments and data analysis described above indicate that the four MATH tandem genes, *TC1a*, are involved in regulating sucrose and starch synthesis. Starch and sucrose accumulation is a complex process of metabolic regulation. AGPase is the first rate-limiting enzyme in starch synthesis and plays a crucial role in plant starch biosynthesis. *APS1* encodes the S subunit of the AGPase complex, and the T-DNA-inserted aps1 mutant lacks starch synthesis [51]. No starch granules were detected in the four-gene deletion mutant, which includes ISA and starch synthase genes (*SS1*, *SS2*, and *SS3*) [52]. This study demonstrated that *TC1a* regulates the accumulation of sucrose and starch (Figure 6). In the leaves of plants overexpressing the *TC1a* gene, the expressions of starch synthesis-related enzyme genes *APS1*, *APL3*, *SS1*, and *GBSS1* were downregulated, while *TC1a* gene expression decreased the expression of these same genes were upregulated (Figure 8), suggesting that *TC1a* inhibits starch synthase gene expression, thereby reducing starch synthesis during the process. The rate of sucrose synthesis is related to the activity of *SPS* [53]. When sucrose is unloaded from the phloem into the cell. It is hydrolyzed into glucose and fructose by the cell wall invertase (*CWINV*) [54]. *cwinv4-2* accumulates lower amounts of starch in the nectarial stomata [55]. The thousand-grain weight of the *cwinv2* mutant is significantly reduced and affects seed development in Arabidopsis [56]. Overexpression of the *TC1a* resulted in the upregulation of sucrose synthases *SPS4F*, *SPS1F*, and *SPP2*, and the downregulation of sucrose invertases *CWINV2*, *CWINV4*, and *APS* in the leaves of plants. Conversely, silencing of the *TC1a* led to downregulation of *SPS4F*, *SPS1F*, and *SPP2*, and upregulation of *CWINV2*, *CWINV4*, and APS1 expression (Figure 8). Based on this, it is proposed that *TC1a* regulates the distribution of carbon sources between sucrose and starch by inhibiting the expression of starch synthase genes and sucrose hydrolases while promoting the expression of sucrose synthase genes.

## 4. Materials and Methods

### 4.1. Plant Material and Growth Conditions

The experimental materials were the Colombian wild-type (WT) *Arabidopsis thaliana* provided by the Key Laboratory of Hunan Provincial on Crop Epigenetic Regulation and Development (Hunan Agricultural University, Changsha, China). Plants were grown in a culture chamber at 25 °C with 50% humidity and a 16/8 h of light/darkness.

### 4.2. Identification of TRAF Proteins

Protein sequences of *A. thaliana* and rapeseed were downloaded from TAIR (https://www.arabidopsis.org/, accessed on 25 April 2024) and BnIR (http://yanglab.hzau.edu.cn/, accessed on 25 April 2024), respectively. Hidden Markov model (HMM) profiles of the MATH domain (PF00917) were retrieved from the Pfam protein database (http://pfam-legacy.xfam.org/, accessed on 25 April 2024), and MATH domain proteins were identified using HMM 3.0 [57], with an E-value < 10^−5^. MATH domain was manually searched using NCBI Batch CD-search tool (https://www.ncbi.nlm.nih.gov/cdd, accessed on 26 April 2024) to confirm MATH domain proteins. Protein sequences were aligned using MUSCLE in MEGA 11.0 to construct a maximum likelihood (ML) tree with 1000 bootstraps [58]. The phylogenetic tree was then visualized and embellished using ITOL (https://itol.embl.de/, accessed on 27 April 2024).

### 4.3. Plasmid Construction

The sgRNAs targeting the second exon of *AtTC1a* were designed using CRISPOR (http://crispor.tefor.net/, accessed on 20 April 2023) (Supplementary Table S1), according to the tandem target sequence synthesis method of tRNA interval [59] and the CRISPR/Cas9 editing system in *Arabidopsis* [60]. A double-target knockout vector was constructed. The gRNA scaffold::tRNA plasmid was synthesized Tsingke (Beijing Tsingke Biotech Co., Ltd., Beijing, China). The primers used were tRNA::sgRNA1-F:5′-CGGAAGACGTGATTAACAAAGCACCAGTGGTCTAGTGG-3′, tRNA::sgRNA1-R:5′-CGGGTCTCGAATACAACAGAGTGCACCAGCCG-3′, gRNA scaffold-tRNA::sgRNA2-F:5′-TAGGTCTCGTATTCTCCGTTGGTTTTAGAGCTAGAAATAGCAAG-3′, gRNA scaffold-tRNA::sgRNA2-R: 5′-TAGAAGACTAAAACATCCGAAGTTCACATCCACCTGCACCAG-3. The PCR products of tRNA::sgRNA1 and gRNA scaffolding-tRNA::sgRNA2 were assembled into tRNA::sgRNA1-gRNA scaffolding-tRNA::sgRNA2 using *Bsa* I and T_4_ DNA Ligase. The reaction program was 5 min at 37 °C, 10 min at 20 °C, followed by 40 cycles, and 60 min at 20 °C. The tRNA::sgRNA1-gRNA scaffold-tRNA::sgRNA2 and pSGR-Cas9-At were digested with *Bbs* I and assembled into pSGR-Cas9-At::sgRNA plasmid. Next, pSGR-Cas9-At::sgRNA and pCAMBA1300 were digested with *Hind* III and *BamH* I, and the pCAMMA1300-Cas9-sgRNA binary expression vector was successfully constructed. Transgenic plants were generated by transformation of *Arabidopsis* (Figure S1) [61].

Using the primers p35S::TC1a-F: 5′-CATGCCATGGATGTTGTCGTCTTCTTCATC-3′ and p35S::TC1a-R: 5′-CACCTAGGTCACGGACTGACCTGACG-3′, we amplified the CDS sequence and cloned it into LB solid culture medium with Kan resistance. The amplified sequence was then assembled into the pFGC5941 plasmid to construct the p35S::TC1a overexpression vector using N*co I*, S*ma I* and T4 DNA Ligase.

The *AtTC1a* promoter sequence was cloned using *BamH* I and *Hind* III into the pBI101 plasmid to construct the TC1apro::GUS plasmid. All primers are listed in Supplementary Table S2.

### 4.4. Screening of Transgenic Arabidopsis thaliana

Genomic DNA was extracted from resistant seedlings using the CTAB method. First, Cas9, HYG, and expression frame were identified. Next, the sgRNA sequence was amplified using TC1a-sgRNA-F:5′-CAAGCCATCTTTCTATATTTCTTGAAGTGAC-3′ and TC1a-sgRNA-R:5′-CTTGTACTTAACCCATAAATTATTATCCACATCG-3′, and the PCR products were cloned into LB solid medium with Kan resistance. The monoclonal colonies were selected, and sequences were detected using primers TC1a-sgRNA-F. The results were analyzed using SnapGene. To obtain *TC1a* homozygous lines, T_1_ transgenic *Arabidopsis* seeds were sown on MS medium and transferred to the culture room once 2–3 leaves had grown. The genomic DNA of the leaves was extracted, the sgRNA sequence was amplified, and the PCR purification were purified for analysis. Meanwhile, primers Cas9-F: 5′ -GAGAGAATGCTGGCCTGC-3′ and Cas9-R: 5′ -GAGCCTTGTTGTAGGCGGACA-3′ were used to confirm plasmid removal. Finally, the *TC1a* homozygous lines were selected for further experimentation.

The *p35S::TC1a* and *TC1apro::GUS* vectors were transferred into WT, and the harvested seeds were labeled as the T_0_ generation. The seeds were sown on MS solid media containing herbicide (20 mg/L) and Kan (50 mg/L) to screen for resistant seedlings. Transgenic *Arabidopsis* plants were identified using the following primers 35S-F:5′-CTTCAAAGCAAGTGGATTGATGTGATATC-3′, p35S::TC1a-R:5′-ACCGGCGGTAAGGATCTGA-3′ and pBI101-TC1apro::GUS-F:5′-GCTTCCGGCTCGTATGTTG-3′, TC1apro::GUS-R: 5′-CGGGATCCTGCCTCCGACGAGATCTG-3′. The relative expression level of *p35S:TC1a* transgenic plants was detected, and T_2_ and T_3_ transgenic plants were further confirmed as homozygous by PCR. All primers used in this study are listed in Supplementary Table S2.

### 4.5. Phenotypic Observation and Statistics

WT and mutant seeds were sown on 1/2 MS solid medium, and their germination was observed. After 10 days, the root length was measured. Simultaneously, the lengths of the stem, silique, and plant height were measured, and the flowering time was recorded.

### 4.6. Sugar Content Determination, Light Treatment, and Starch Staining

Starch and sucrose content in the rosette leaves at both the seedling and maturation stage were measured using visible spectrophotometry. For detailed steps, refer to the instructions of the starch [62], sucrose [63], and soluble sugar [64,65] kit instructions of Solarbio (Beijing Solarbio Science & Technology Co., Ltd., Beijing, China).

After 4 weeks of growth, the plants were exposed to different light durations. First, plants were grown under dark conditions for 48 h to deplete starch, followed by exposure to light for 0 h, 4 h, 8 h, 12 h and 16 h. The samples were then used for starch staining, and the remaining samples were stored at −80 °C for carbohydrate content analysis.

For starch staining, samples were immersed in 80% (*v*/*v*) ethanol and decolorized in a drying oven at 80 °C. The alcohol was replaced 2–3 times until the leaves were fully decolorized. After decolorization, the materials were placed in Lugol solution (5% iodine *w*/*v* and 10% potassium iodide *w*/*v*) and stained in the dark for 30 min [66]. After staining, the sample was washed with water three times to remove excess Lugol and photos were taken for observation.

### 4.7. Histochemical Staining

Different tissue from transgenic plants, including rosette leaves, cauline leaves, roots, stems, flowers, and siliques, were stained using histochemistry methods. For the specific staining method, please refer to the GUS Stain Kit of Solarbio Biotechnology (Beijing Solarbio Science & Technology Co., Ltd., Beijing, China).

### 4.8. Total RNA Extraction and RT qPCR Analysis

A Promega RNA Extraction Kit (Promega, Beijing, China) was utilized to extract RNA. The cDNA was obtained from 1 μg RNA using Thermo Fisher Scientific Reverse Transcription Kit (Thermo Fisher Scientific, Shanghai, China). RT-qPCR was performed using SYBR Green fluorescence in the CFX96TM Real-Time system, with the program set as follows: initial denaturation at 95 °C for 30 s, denaturation at 95 °C for 10 s, followed by 40 cycles, annealing and extension 95 °C for 30 s, followed by 40 cycles. Each reaction was performed in triplicate with three biological replicates and technical repetitions. The 2^−∆∆Ct^ method was used to determine relative expression, with *ACTIN 2* as the reference gene [67]. Ordinary one-way ANOVA in GraphPad Prism 9.0.0 was used for data analysis. NCBI (https://www.ncbi.nlm.nih.gov/tools/primer-blast, accessed on 2 January 2024) was used to design RT-qPCR primers and specific amplification detection (Supplementary Table S2).

### 4.9. Transcriptomic Analysis

Total RNA was extracted from *TC1a* knockout lines, overexpression lines, and WT leaves. RNA concentration, purity, and integrity were assessed by agarose gel electrophoresis, and then, the RNA seq library was constructed. Sequencing was performed on the Illumina HiSeq6000 sequencing platform (Illumina, San Diego, CA, USA) with paired-end (150 bp) reads. Raw data were quality-controlled using fastp [68]. STAR [69] software was used to align clean data with the reference genome. Transcriptome data quality was evaluated using RSeQC [70], and transcripts were then merged with StringTie [71] and GffCompare [72] to generate comprehensive data. Transcript expression levels were estimated using StringTie [71], and FPKM (fragments per kilobase of transcript per million fragments mapped) values were calculated. DEGs were identified using DESeq2 [73], with a threshold of VIP > 1 and *p*-value < 0.05. Finally, EggNOG [74] was used for perform GO (gene ontology) [75] and KEGG [76] analysis.

### 4.10. Targeted Metabolomics Analysis

Sample preparation and metabolite detection were provided by Sanshubio (Sanshubio, Shanghai, China). To prepare the samples, 100 mg were placed in an EP tube with 700 μL of 80% ethanol solution. The mixture was shaken at 50 °C for 2 h, followed by the addition of 700 μL of water for dilution, centrifuged at 10,000 rpm for 3 min and the supernatant was diluted several times. A 10 μL sample was injected into a CarboPac™ PA1 (250 × 4.0 mm) liquid chromatography column, and the sugar components were analyzed using an electrochemical detector. Raw data were processed using Chromeleon to calculate the absolute content of metabolite. After standardizing the raw data using pareto scaling, Orthogonal partial least-squares discriminant analysis (OPLS-DA) was performed. The VIP (variable importance in project), fold change (FC), and *p*-value were calculated to assess differences in metabolite content between the samples. DAMs were screened with *p* value < 0.05 and VIP ≥ 1 [73].

### 4.11. Correlation Analysis Between Transcriptomics and Metabolomics

All DEGs and DAMs related to the starch–sucrose metabolism pathway were selected for correlation analysis using the Pearson correlation method, with Pearson correlation ≥ 0.8, *p*-value ≤ 0.05.

## 5. Conclusions

A total of 81 TRAF proteins were identified in rapeseed, and 200 in *Arabidopsis*. *TC1a* overexpressing and knockout homozygous plants were also generated. Overexpressing lines showed significant dwarfism, small leaves, slower germination rate, and delayed flowering, while knockout lines showed no significant phenotypic changes with significant changes in sucrose and starch content being observed. Overexpression of *TC1a* induced the expression of sucrose synthase (*SPS1F*, *SPS4F* and *SPP2*) and starch hydrolases (*BAM1*, *BAM5*, *BAM6* and *AMY1*), leading to sucrose accumulation. In contrast, the knockout strain promoted the expression of starch synthase (*SS1*, *APS1*, *GBSSI*) and increased starch content.

## Figures and Tables

**Table 1:** VIP and *p*-value of DAMs in overexpressing, knockout plants, and WT.

WT_vs_TC1a-OE		
Index	VIP	*p*-Value
Trehalose	1.012117949	6.15 × 10^−7^
Sucrose	1.012194433	3.52 × 10^−6^
Galactose	1.010228328	2.65 × 10^−5^
Glucuronic acid	1.0110363	3.31 × 10^−5^
Arabinose	1.00790809	3.81 × 10^−5^
Gluconic acid	1.012432001	4.19 × 10^−5^
Raffinose	1.012516777	9.84 × 10^−5^
Fructose	1.012275746	0.000123904
Stachyose	1.012243583	0.000174401
Ribonic acid	1.012160363	0.00028317
Maltose	1.011556905	0.000393272
myo-Inositol	1.006634873	0.002871083
Glucose	1.00498228	0.003347305
Rhamnose	1.004251667	0.003838229
Sorbitol	0.999695661	0.004386046
Lactose	1.000924743	0.004652827
Mannose	0.987177024	0.009775609
Xylose	0.957467558	0.025824305
Fructose 6-phosphate	0.906009032	0.039504685
**WT_vs_TC1a**		
Trehalose	0.960339482	0.048561881
Glucose	1.008616085	0.005067896
Fructose	1.002897589	0.014205952
Sucrose	1.0300611	0.001591717
Maltose	0.996800999	0.019496026

## Data Availability

The transcriptomic sequencing data can be downloaded from the BioProject database (accession number: PRJNA1145560).

## Supplementary Materials

The following supporting information can be downloaded at: https://www.mdpi.com/article/10.3390/plants13233402/s1.
