# Supplementary material for: Transcriptome and Metabolome Analyses Reveal the Regulatory Mechanism of TC1a in the Sucrose and Starch Synthesis Pathways in Arabidopsis thaliana
Source: Plants (Basel). 2024 Dec 4;13(23):3402. doi: 10.3390/plants13233402 (PMC11644801; doi:10.3390/plants13233402)
Supplement: Supplementary file 1 [file plants-3274206-figures.pdf › plants-3274206-figures.pdf]

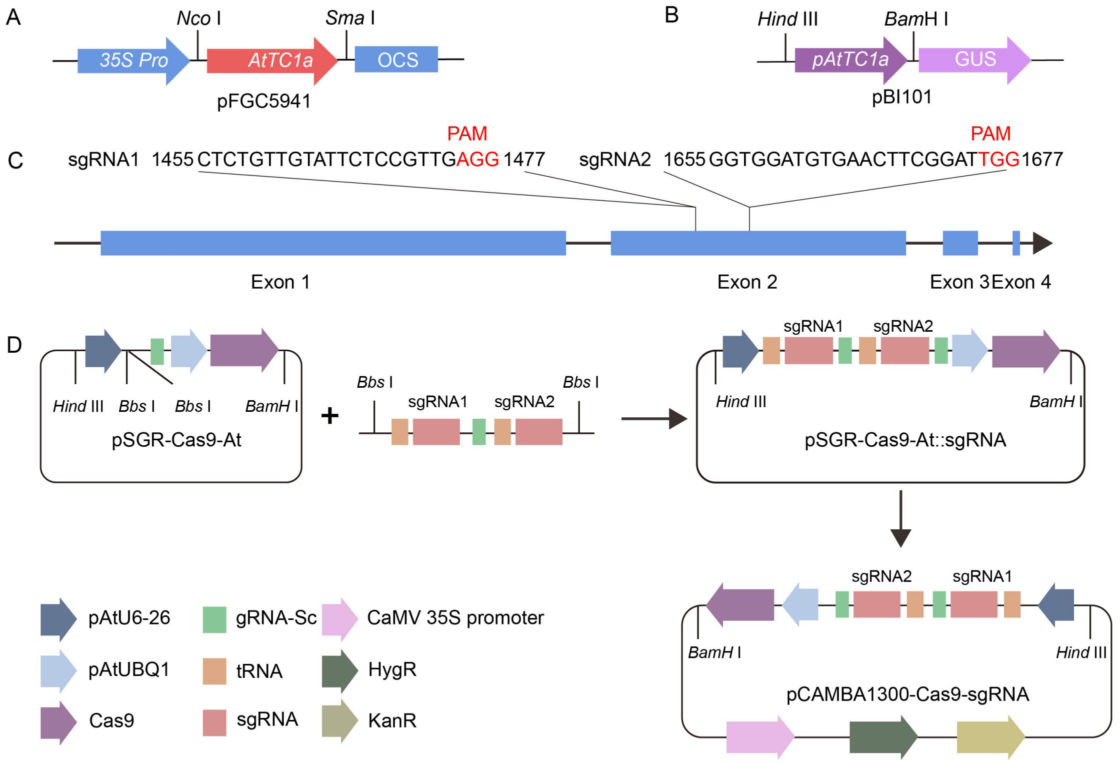

Figure S1. Schematic diagram of expression vector construction. A,B : Overexpression and GUS expression vector construction. C: Selection sites for sgRNAs of *TC1a*. D: Construction of CRISPR/Cas9 Knockout Vector.

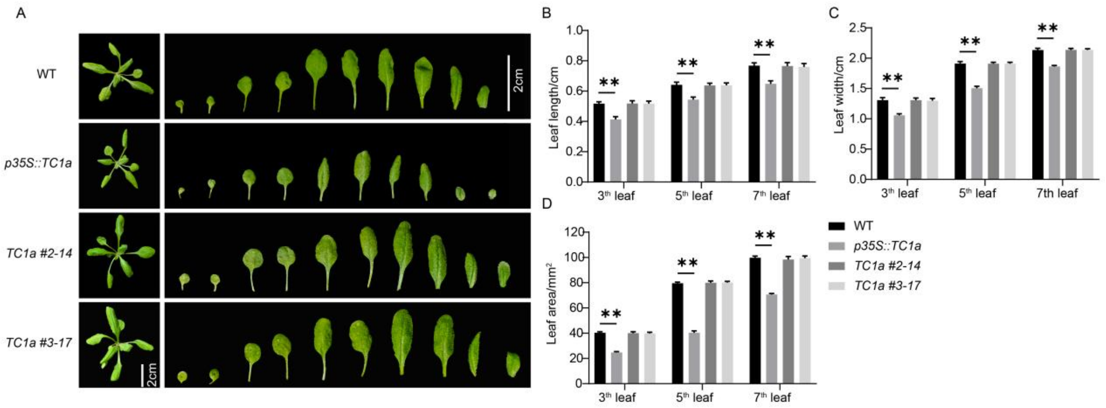

Figure S2. Comparison and statistics of leaf size between WT and transgenic lines. A: 10 leaves stage *Arabidopsis*. B: Statistical analysis of the length, width and leaf area of 3th, 5th and 7th leaves.

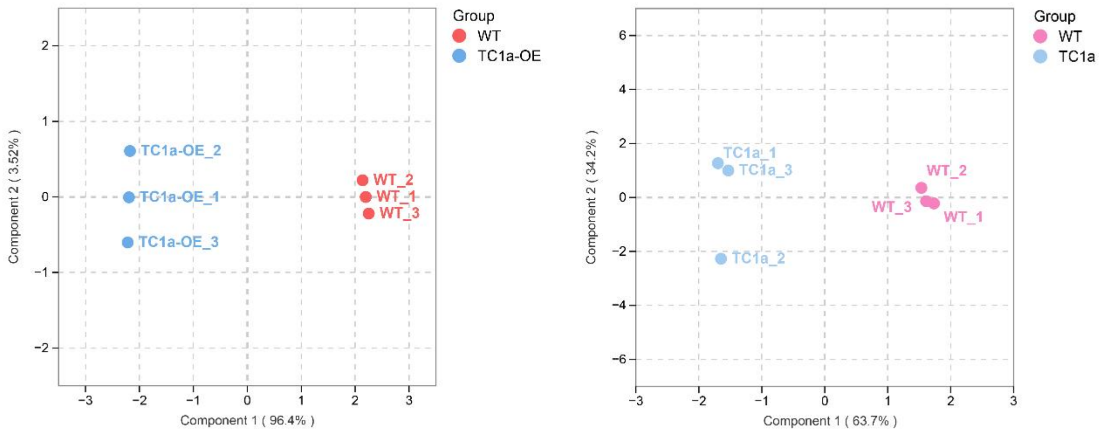

Figure S3. OPLS-DA analysis of metabolites between samples.

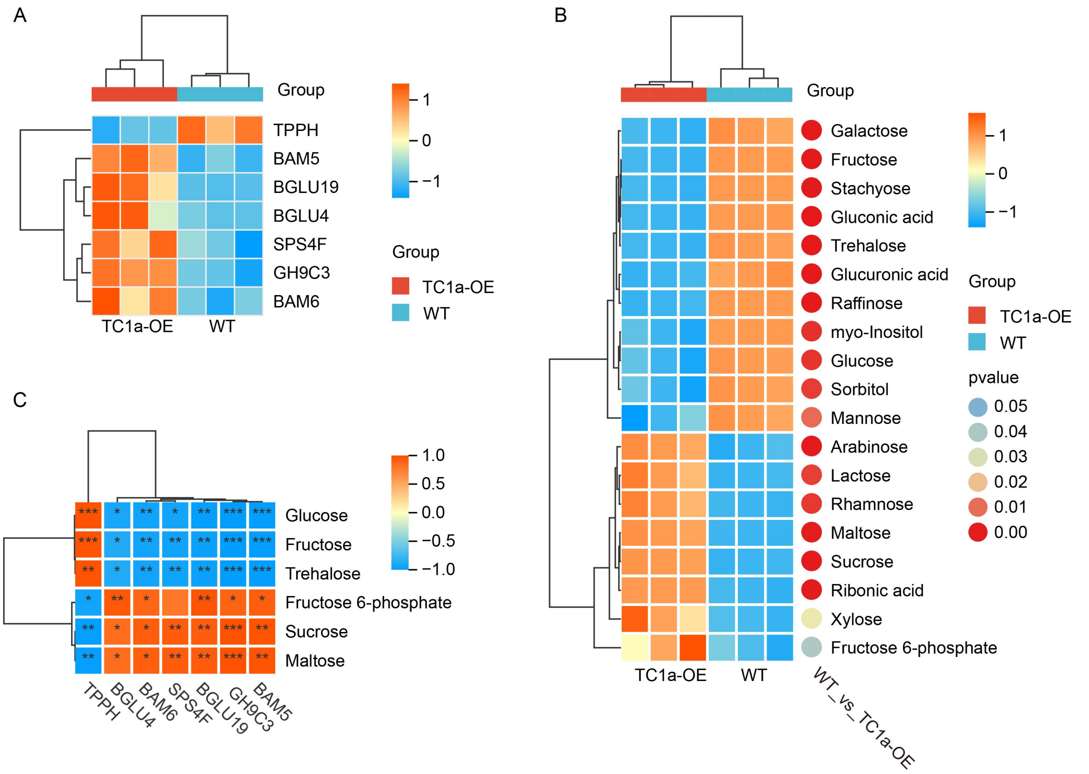

Figure S4. Transcriptome and metabolome analysis in WT_vs_TC1a-OE. A: DEGs clustering heatmap related to sucrose and starch metabolism pathways. B: DAM clustering heatmap. C: DEGs and DAMs correlation analysis. The data were normalized by Z-score. G,N: Set Pearson correlation ≧ 0.8 and p value ≦0.05 for correlation analysis.

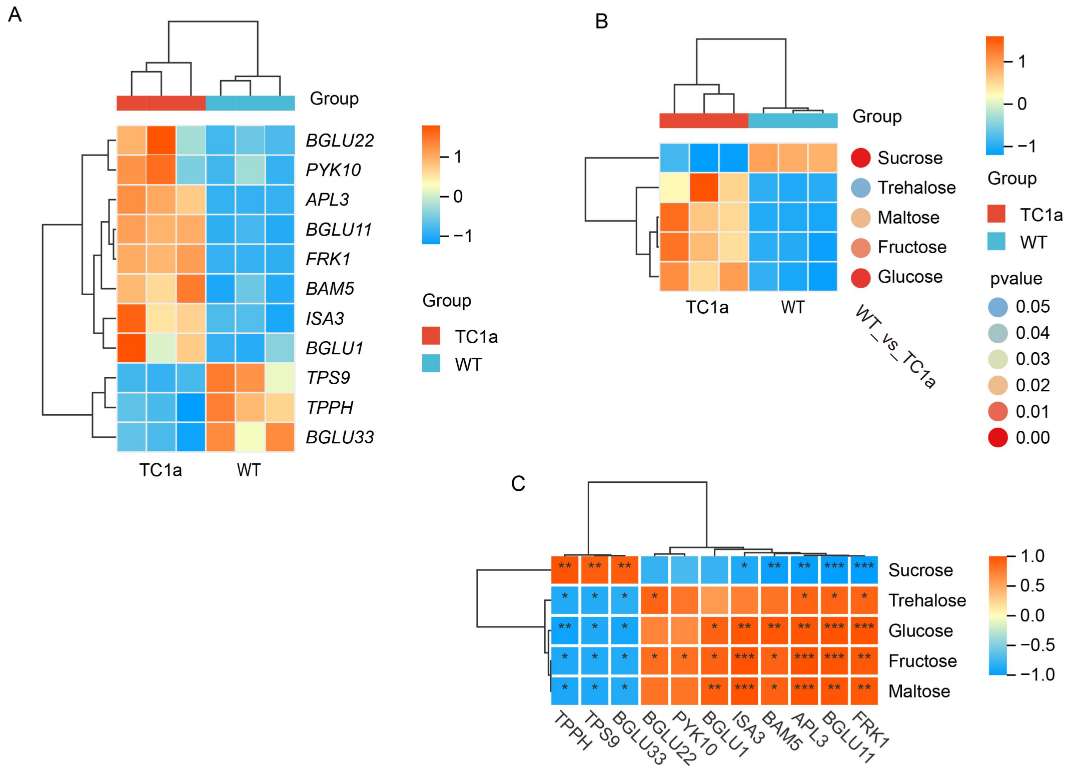

Figure S5. Transcriptome and metabolome analysis in WT_vs_TC1a. A: DEGs clustering heatmap related to sucrose and starch metabolism pathways. B: DAM clustering heatmap. C: DEGs and DAMs correlation analysis. The data were normalized by Z-score. G,N: Set Pearson correlation ≧ 0.8 and p value ≦0.05 for correlation analysis.
